# Biological Response to a Novel Hybrid Polyoligomer: *in vitro* and *in vivo* Models

**DOI:** 10.17691/stm2020.12.6.05

**Published:** 2020-12-28

**Authors:** A.E. Bokov, A.A. Bulkin, D.V. Davydenko, N.Yu. Orlinskaya, M.N. Egorikhina, Yu.P. Rubtsova, I.N. Charykova, R.S. Kovylin, V.V. Yudin, S.A. Chesnokov, A.G. Morozov, S.G. Mlyavykh, D.Ya. Aleynik

**Affiliations:** Head of the Department of Oncology and Neurosurgery, University Clinic; Privolzhsky Research Medical University, 10/1 Minin and Pozharsky Square, Nizhny Novgorod, 603005, Russia;; Neurosurgeon, University Clinic; Privolzhsky Research Medical University, 10/1 Minin and Pozharsky Square, Nizhny Novgorod, 603005, Russia;; Researcher, Pathological Anatomy Group, University Clinic; Privolzhsky Research Medical University, 10/1 Minin and Pozharsky Square, Nizhny Novgorod, 603005, Russia;; Professor, Head of Pathological Anatomy Department, University Clinic; Privolzhsky Research Medical University, 10/1 Minin and Pozharsky Square, Nizhny Novgorod, 603005, Russia;; Leading Researcher, Laboratory of Regenerative Medicine, Research Institute of Experimental Oncology and Biomedical Technologies; Privolzhsky Research Medical University, 10/1 Minin and Pozharsky Square, Nizhny Novgorod, 603005, Russia;; Researcher, Laboratory of Regenerative Medicine, Research Institute of Experimental Oncology and Biomedical Technologies; Privolzhsky Research Medical University, 10/1 Minin and Pozharsky Square, Nizhny Novgorod, 603005, Russia;; Physician, Biotechnology Laboratory, University Clinic; Privolzhsky Research Medical University, 10/1 Minin and Pozharsky Square, Nizhny Novgorod, 603005, Russia;; Researcher, Laboratory of Photopolymerization and Polymer Materials; G.A. Razuvaev Institute of Organometallic Chemistry, Russian Academy of Sciences, 49 Tropinina St., Nizhny Novgorod, 603137, Russia; Junior Researcher, Laboratory of Photopolymerization and Polymer Materials; G.A. Razuvaev Institute of Organometallic Chemistry, Russian Academy of Sciences, 49 Tropinina St., Nizhny Novgorod, 603137, Russia; Leading Researcher, Laboratory of Photopolymerization and Polymer Materials; G.A. Razuvaev Institute of Organometallic Chemistry, Russian Academy of Sciences, 49 Tropinina St., Nizhny Novgorod, 603137, Russia; Senior Researcher, Applied Research Laboratory; G.A. Razuvaev Institute of Organometallic Chemistry, Russian Academy of Sciences, 49 Tropinina St., Nizhny Novgorod, 603137, Russia; Director of the Institute of Traumatology and Orthopedics; Privolzhsky Research Medical University, 10/1 Minin and Pozharsky Square, Nizhny Novgorod, 603005, Russia;; Senior Researcher, Laboratory of Regenerative Medicine, Research Institute of Experimental Oncology and Biomedical Technologies Privolzhsky Research Medical University, 10/1 Minin and Pozharsky Square, Nizhny Novgorod, 603005, Russia;

**Keywords:** oligoester di(meth)acrylate, polylactide, hybrid porous polyoligomer, bone defect model, tissue remodeling, cytotoxicity, biocompatibility

## Abstract

**The aim of the study** is to evaluate biocompatibility of a novel hybrid polyoligomer in *in vitro* and *in vivo* models.

**Materials and Methods.:**

Cytotoxicity of the material was investigated using the MTT assay with human dermal fibroblasts as test cultures. To study direct interaction of the hybrid polyoligomer with cells, the fibroblasts were cultured on the polymer samples for 96 h, the cultures were assessed every 24 h using fluorescence microscopy. To study the tissue reaction in the area of contact with the donor bed and the morphological features of the implanted sample restructuring, a case-control study was performed using a rabbit model. Samples of hybrid polyoligomer were implanted into the bone defect formed in the left iliac crest in 10 rabbits. In the control group, the prepared allograft samples were transplanted into similar defects in 10 animals. The rabbits were sacrificed 4 and 8 weeks after the operation. The standard morphological methods with hematoxylin and eosin staining and immunohistochemical Ki-67 proliferation marker evaluation were used to assess the state of tissues in the defect area.

**Results.:**

The results demonstrate that the hybrid polyoligomer is not cytotoxic (cytotoxicity score 0–1), cells adhere well to its surface, retain their viability and typical morphology throughout the entire observation period. No negative impact of material implantation on the health state and behavior of animals was detected. Morphological examination showed the absence of inflammatory changes, formation of thin-walled capillary vessels, and considerable proliferative activity of mesenchymal cells in the defect area, even though it was more intense than in the control group.

**Conclusion.:**

No inflammation signs were detected by 8^th^ week of the experiment. It was defined that new bone was beginning to form. The results of analysis support the conclusion that the developed hybrid materials are prospective for further research as potential bone substitute.

## Introduction

The problem of bone defects repair caused by various diseases, injuries, or complex surgical interventions remains actual. There is considerable lack of donor material for auto- and allografts [1–4], and various problems related to the use of xenogenic biomaterials [5, 6], therefore, biocompatible polymers attract increasingly greater attention. Due to such benefits as low weight, the possibility to use monomers and/or polymers as ink for 3D printing in manufacturing personalized implants, obtaining and storing any required amount of material and standard products, the opportunity to vary the specified parameters in a wide range, polymers have obvious advantages over other materials. By now, a large variety of polymers used in medicine has been described. The most promising of those biodegradable polymers are materials based on polylactides and their copolymers, and implants made of biostable materials, which may have an invariable rigid mesh structure providing an opportunity for adhesion and subsequent functioning of recipient cells [7–9].

Porosity is known to be one of the key requirements for bone substitute materials along with strength, modelability, osteoconductivity, and osteoinduction. The system of interconnected pores should provide effective circulation of tissue fluid with subsequent ingrowth of small blood vessels and recruitment of recipient cells into the transplanted constructs. Porous cross-linked polymers (three dimensional polymer network) are considered promising options for creating stable implants with the required mechanical properties and pore volume. Porous cross-linked polymer products of a given geometry made of them can be obtained by making use of recent advances in additive manufacturing [10]. Polymers based on oligoester di(meth)acrylates are nontoxic; therefore, they are widely used in filling materials, “bone cement”, and contact lenses [11–13].

We have obtained porous polyoligomeric matrices demonstrating the absence of cytotoxicity, high adhesion, and proliferation of mesenchymal stem cells on their surface [14, 15]. The developed technology for manufacturing the material has enabled obtaining a porous polyoligomer with a system of open interconnected pores of various sizes and different porosities, which makes it possible to bring its characteristics closer to the porosity of various bone segments. The use of biocompatible catalytic systems based on alkaline earth elements (magnesium, calcium) made it possible to obtain atactic polylactide by D-, L-lactide ring-opening polymerization, which, as found in *in vitro* experiments, demonstrates the absence of cytotoxicity towards human dermal fibroblasts. Besides, soluble polymer degradation products released during hydrolysis act as growth stimulators, providing stable cell proliferation [16].

It also seemed advantageous to obtain material combining the properties of polyoligomer and polylactide, which could improve the characteristics of the material. To solve this problem, we developed a hybrid polyoligomer (HPO) material based on polyoligomer and polylactide with 65–70% porosity and pore size range of 5 to 20 μm. First of all each new material planned for medical use should be tested on biological models, which predetermined the aim of this investigation.

**The aim of the study** is to evaluate biocompatibility of the developed hybrid polyoligomer in biological models *in vitro* and *in vivo*.

## Materials and Methods

The investigation protocol was approved by the Ethics Committee and approved by the Academic Council of Privolzhsky Research Medical University (Nizhny Novgorod, Russia). All procedures involving animals were carried out in a vivarium according to the requirements of the European Convention for the Protection of Vertebrate Animals used for Experimental and Other Scientific Purposes (Strasbourg, 2006) and in accordance with the Order of the Ministry of Health and Social Development of the Russian Federation No.199n “On Approval of the Rules of Good Laboratory Practice” (2016).

### Test samples.

The HPO material for research was obtained from a porous polyoligomeric matrix by applying a polylactide (molecular weight — 2·10^4^ Da) layer on the pore surface. The pore characteristics of the material were determined by mercury porosimetry using the PASCAL EVO 140/440 ULTRA MACRO porosimeter (Thermo Fisher Scientific, USA). The porosity of the material was 68.1%, the average pore size was 10.7 μm. The samples were white opaque plates. Sample sizes corresponded to the size of the experimental bone defect in animals.

### Description of the test culture.

At the first stage of work, interaction of the HPO material samples with the cell culture was investigated using human dermal fibroblasts as test cultures. The cultures were sterile and tested for mycoplasma and viral contamination by PCR. Before entering the experiment, the culture cells were morphologically homogeneous, predominantly fusiform, and formed a subconfluent monolayer. Cell viability in cultures was 98–99%. The proportion of cells with the CD90^+^, CD105^+^, CD73^+^, CD10^+^, CD45^–^, CD34^–^, CD14^–^, CD HLA-DR^–^ phenotype typical of mesenchymal cells was 99%. Cultures of the 4^th^ passage were used for the study.

### Evaluation of cytotoxicity of the material.

Cytotoxicity was evaluated using the MTT assay. This test is based on the ability of living cells to reduce yellow 3-(4,5-dimethylthiazol-2-yl)-2,5-tetrazolium bromide (MTT) to purple intracellular MTT-formazan crystals, soluble in isopropanol or dimethyl sulfoxide. The amount of product reduced is measured photometrically at 540 nm. A statistically significant decrease in the optical density of test samples in comparison with the control samples, recorded on a plate reader, makes it possible to evaluate the cytotoxic effect of the test substance on cells [17].

During the experiments, test samples were immersed in α-MEM medium containing antibiotics and 2% fetal calf serum on days 1 and 7, then incubated under standard conditions. After 1 and/or 7 days, the extract above the samples was collected. The test culture was prepared at the same time. For this, fibroblasts at a concentration of 1·10^5^ were seeded in wells of a flat-bottom 96-well plate in α-MEM medium with antibiotics and 10% inactivated fetal calf serum and cultured under standard conditions for 3 days.

After this period, the growth medium above the cells was replaced with the extract from the samples and its dilutions (1:1; 1:2; 1:4; 1:8). After 24 h, the medium in the wells was replaced with MTT solution, and the cells were incubated with MTT for another 4 h. MTT solution was prepared in Hanks’ balanced solution at a concentration of 1 mg/ml. After 4 h of incubation, the supernatant was carefully aspirated, dimethyl sulfoxide (200 μm) was added, and the optical density was recorded at 540 nm on the Sunrise analyzer (Tecan Austria GmbH, Austria).

The relative growth rate (RGR) was calculated using the following formula:

*RGR* (%) = *Average optical density in the tested culture / Average optical density in control* **·** 100.

RGR intensity was determined based on cytotoxicity rank scale [18].

### Assessment of cell adhesion on the material surface.

To assess adhesion, a suspension of the 4^th^ passage fibroblasts at a concentration of 2·106/ml was inoculated on the surface of the samples. Plates with samples were placed in a CO_2_ incubator at 37°C and 5% CO_2_ in a humid atmosphere, cultured for 96 h, culture growth was monitored every 24 h. The growth medium was changed after 48 h. After 24, 48, and 96 h of cultivation, some of the samples were taken for investigation by fluorescence microscopy, which was performed on the Cytation 5 Cell Imaging Multi-Mode Reader (BioTek, United States).

Vital staining of nuclei in cells adhered to the material was performed using Hoechst 3334 fluorochrome (BD Pharmingen, USA) with high specificity for a double-stranded DNA molecule (excitation wavelength 377 nm, emission wavelength 447 nm). Calcein (Calcein AM; BD Pharmingen; excitation wavelength 469 nm, emission wavelength 525 nm) was used to mark living cells and characterize their morphology. Fluorochrome staining was performed according to the manufacturer’s protocols.

### Experimental model in vivo.

Chinchilla rabbits were used as an animal model. The choice of this model was determined by the fact that rabbit bones have characteristics similar to the biomechanical characteristics of human bones [19]. The size of the animal is sufficient for implantation of sizable grafts in order to study the tissue reaction in the area of “graft–donor bed” interface and the morphological features of sample restructuring. To study biocompatibility of the HPO material, a rabbit iliac crest defect model was selected. This decision made it possible to minimize the trauma of access to this bone structure and to simulate a bone defect sufficient for studying osseointegration of bone substitute material. To replace the defect in the control group of animals, samples were prepared from rabbit allo-bone, because allografts maximally correspond to the recipient’s own bone in their anatomical, physicochemical characteristics, and the content of morphogenetic proteins [20]. To prepare the allograft, the ilium of the euthanatized intact animal was isolated and completely separated from soft tissues. Next, the bones were fixed in neutral 10% formalin solution for a month. After a month, specimens were cut from the prepared bone, corresponding in shape and size to that of the experimental bone defect, thoroughly washed with sterile saline, and sterilized.

### Experimental procedure.

The experiment was carried out on 20 adult male rabbits aged 6–8 months, weighing 2900 to 3500 g, without atherosclerotic vascular lesions. For anesthesia, a mixture of 1.0 ml of XylaVET (Pharmamagist Ltd, Hungary) and 1.0 ml of Zoletil (Virbac Sante Animale, France) was administered intramuscularly; after pre-operative hair removal and shaving and shaving, the surgical field was treated with the Avansept antiseptic. The access was performed with a 3 cm linear incision in the projection of the left iliac crest. Then the crest section was skeletonized over a length of 1.5 cm. A bicortical bone defect of 1.0×0.5×0.5 cm with flat surfaces was formed using the Verticem V + 8g cutter (Synthes GmbH, USA). Allografts were fitted to this defect in animals of the control group, while implants from the HPO material were implanted in the experimental group. The samples were fitted tightly to provide the maximum contact area in the defect region, after which they were secured with bone suture using VICRYL 2-0. Before completing the surgery, a thorough hemostasis was carried out and a layer-by-layer wound closure was performed.

During the observation, the behavior of the animals, changes in their appetite, weight gain, and the state of tissues in the surgical wound area were assessed. All animals had free access to water and received a sufficient amount of food.

Five animals of the experimental and control groups were sacrificed after 4 weeks (30 days), the rest were euthanatized 8 weeks (60 days) after the operation. For euthanasia, anesthetics in a lethal dose (3 times the dose required to provide anesthesia) were introduced. After euthanasia, the experimental defect area of the left ilium was skeletonized in each animal of both experimental and control groups, a fragment of the bone was excised in the experimental wound area and transferred for morphological examination.

### Morphological examination.

The obtained material was fixed in neutral 10% formalin solution. The bone tissue was decalcified in an acid-free solution. Standard histologic diagnosis was performed using the Excelsior ES apparatus (Thermo Scientific, USA). After the diagnosis, paraffin blocks were made using the HistoStar embedding workstation (Thermo Scientific). 4–6 μm thick sections were obtained using the Microm HM 325 microtome (Thermo Scientific), stained with hematoxylin and eosin using the Gemini AS staining station (Thermo Scientific). For morphometric processing and creation of a video archive of the obtained material, the Leica 2500 microscope (Leica Biosystems, Germany) was used, field lens ×4, ×10, ×20, ×40, ×100, eyepiece lens ×10.

### Immunohistochemistry.

 To assess proliferation activity in the area of material implantation, immunohistochemical examination was performed using the Ki-67 proliferation marker. Examination protocol included incubation of sections with primary antibodies — monoclonal mouse antibodies to Ki-67 (clone MIB-1; Dako, Denmark) and secondary antibodies Goat anti-Mouse IgG H+L, Alexa Fluor 488 (Abcam, USA). To improve visualization, cell nuclei were stained with Hoechst 3334 dye (BD Pharmingen, USA). After obtaining the preparations, the proportion of Ki-67^+^ cells was counted.

### Statistical analysis.

The study results were processed by means of nonparametric statistics using Mann–Whitney U-test and Wilcoxon test for paired comparisons; correlation analysis was performed by means of Spearman’s rank correlation coefficient using the Statistica 6.0 software package. The results were presented as M±m, where M is the arithmetic mean, m is the standard error of the mean.

## Results and Discussion

### Evaluation of cytotoxicity of HPO samples.

HPO samples demonstrated the absence of cytotoxicity (rank 0–1 on the scale) [18] (see the Table).

**Table:** HPO cytotoxicity (M±m)

Material	Parameters	Extraction time	
		Day 1	Day 7
Control (n=8)	Optical density	0.39±0.01	0.47±0.02
	RGR (%)	100	100
	Cytotoxicity level	0	0
Extract (n=8)	Optical density	0.37±0.01	0.42±0.01
	RGR (%)	95	89
	Cytotoxicity level	1	1
Extract (n=8) 1:1	Optical density	0.37±0.01	0.47±0.02
	RGR (%)	95	100
	Cytotoxicity level	1	0
Extract 1:2 (n=8)	Optical density	0.33±0.01	0.50±0.01
	RGR (%)	85	106
	Cytotoxicity level	1	0
Extract 1:4 (n=8)	Optical density	0.39±0.01	0.47±0.01
	RGR (%)	100	100
	Cytotoxicity level	0	0
Extract (n=8) 1:8	Optical density	0.35±0.01	0.49±0.01
	RGR (%)	90	104
	Cytotoxicity level	1	0

According to the cytotoxicity rank scale [18], levels 0 and 1 correspond to the absence of cytotoxicity in the material, while levels 2–5 show varying degrees of toxicity. The developed HPO material was intended for use in conditions of prolonged contact with blood and tissues, therefore the observed absence of cytotoxicity served as the necessary condition for further use of the material.

**Figure 1:**
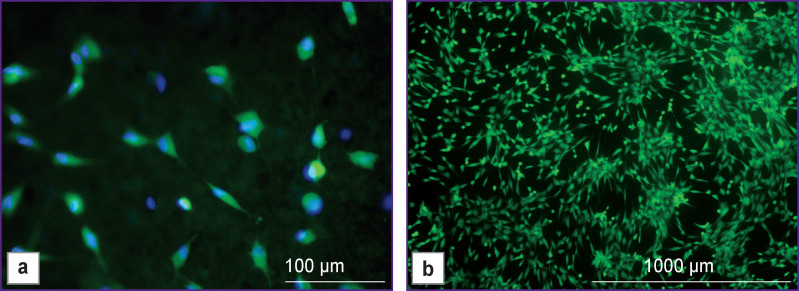
Human dermal fibroblasts on the surface of HPO material: (a) culturing for 24 h: fusiform cells with visible processes; (b) culturing for 96 h: subconfluent monolayer of fibroblasts formed by fusiform cells. Fluorescence microscopy; cell nuclei are colored blue (fluorochrome Hoechst 3334 (BD Pharmingen); excitation wavelength 377 nm, emission wavelength 447 nm); green staining: cytoplasm of viable cells (fluorochrome Calcein AM (BD Pharmingen); excitation wavelength 469 nm, emission wavelength 525 nm); (a) ×200; (b) ×40

After being cultured directly on test material samples for 24 h, fibroblasts adhered well and proliferated on the sample surface (Figure 1 (a)), and after 96 h they already formed a sub-confluent monolayer (Figure 1 (b)).

The use of fluorescence microscopy made it possible to clearly visualize not only marked adhesion of cultured fibroblasts directly on the surface of the studied samples but also to show the morphology of cells and their preserved viability during the entire observation period.

Thus, the study has demonstrated the absence of cytotoxicity of the HPO material thereby proving its longterm benefits for further biomedical use.

### The state of experimental animals during observation.

All experimental animals tolerated the applied surgery very well, remained active in the period of postoperative observation, and gained weight. No infectious diseases, general or local complications were observed.

### Morphological parameters of the bone in the implantation area.

The results of carried out morphological examination showed that processes of bone defect healing in experimental and control animals occurred in accordance with common patterns, including the phases of post-traumatic changes in tissue elements, regeneration, and adaptive remodeling. There were no visible signs of inflammatory reactions in response to introduction of implanted materials in either the control group or the experimental group.

**Figure 2:**
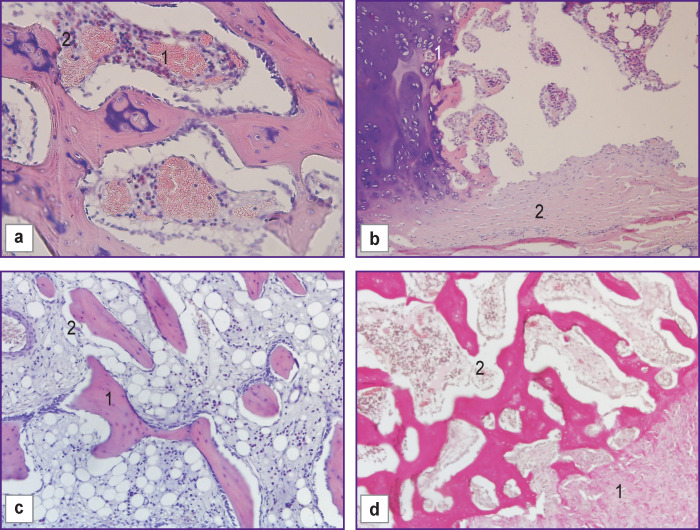
Morphological changes in the control group with allograft, day 30 of the experiment: (a) in the allograft location area there is marked vascular congestion, hemorrhages (*1*), proliferation of osteoblasts and overgrowth of mesenchymal cells (*2*); (b) formation of immature bone tissue with the presence of cartilaginous elements (*1*) and a connective tissue capsule around the defect (*2*); (c) proliferation of osteoblasts (*1*) and overgrowth of mesenchymal cells (*2*); (d) formation of a connective tissue capsule (*1*) around the defect area (*2*); (a)–(c) hematoxylin and eosin staining; (d) Van Gieson staining; ×100

On day 30 of the experiment, in the control group (n=5) with the use of allograft, the histologic pattern in the bone defect area showed indirect perichondral osteogenesis taking place along with the formation of bone plates, proliferation of osteoblasts and mesenchymal cells. A connective tissue capsule was also observed to form around the implant area (Figure 2).

**Figure 3:**
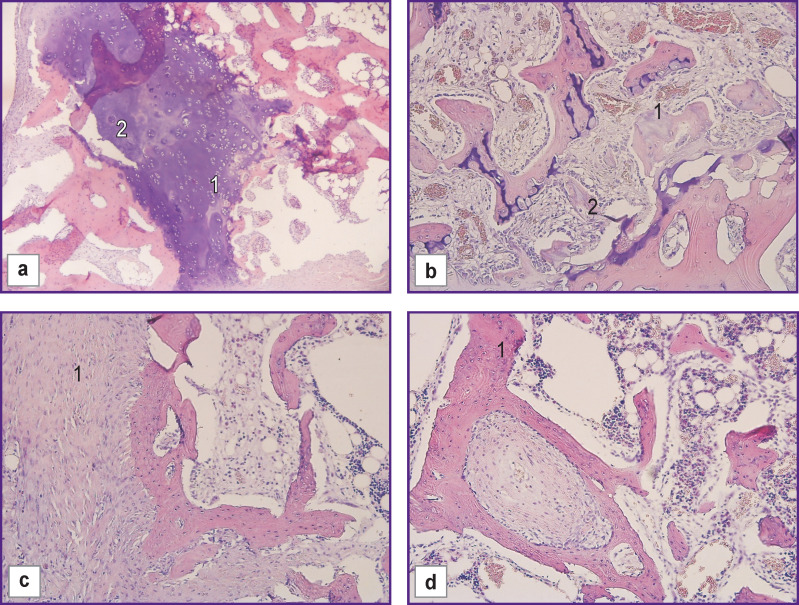
Morphological changes in the control group with allograft, day 60 of the experiment: (a) formation of immature bone tissue with the presence of cartilaginous elements (*1*) and newly formed bone (*2*); (b) formation of immature bone tissue with the presence of cartilaginous elements (*1*), formation in the lacunae of a newly formed bone, consisting of osteoblasts and osteocytes (*2*); (c) proliferation of the connective tissue capsule around the defect area (*1*); (d) formation of a newly formed bone (*1*); hematoxylin and eosin staining; ×100

After 60 days, immature bone tissue consisting of osteoblasts and osteocytes with the presence of cartilaginous elements was formed in the control group (n=5). The connective tissue capsule continued growing around the defect area (Figure 3).

There were some differences in reparative bone tissue regeneration when using HPO implants. After 30 days of the experiment, formation of the connective tissue capsule around the implant area was accompanied by growth of soft fibrous connective tissue around the fine-granular structures of the material with a large number of thin-walled newly formed vessels of the capillary type. There was proliferation of osteogenic cells, their transformation into osteoblasts, and formation of new bone tissue in the form of bone trabeculae, with capillary vessels forming in the inter-trabecular spaces. The bony lacunae contained fragments of the HPO.

**Figure 4:**
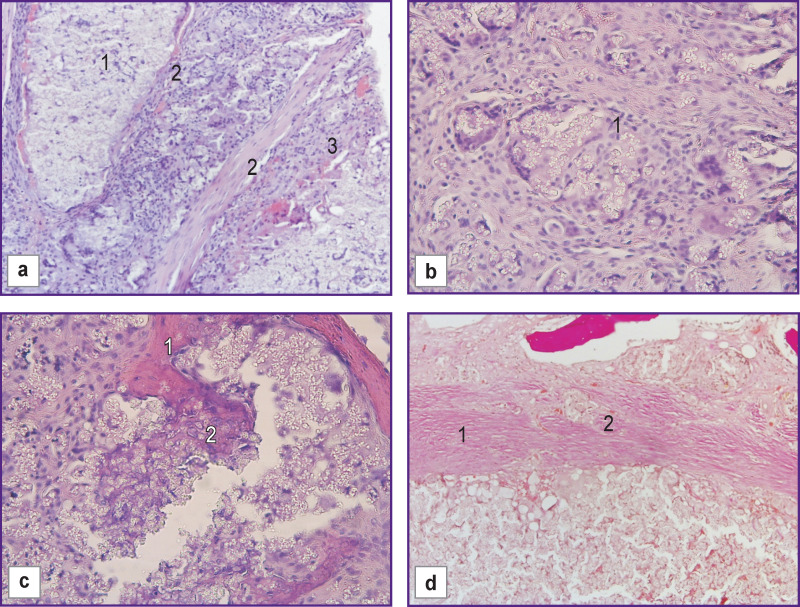
Morphological changes in the experimental group with HPO, day 30 of the experiment: (a) formation of mature connective tissue (*2*), granulation tissue, newly formed vessels (*3*) around the implant zone (*1*); (b) diffuse growth of connective tissue around the implant structures and blood vessels beginning to form (*1*); (c) formation of immature bone tissue intimately connected with the structures of the implant (*2*) in the areas of connective tissue fiber growth (*1*); (d) mature fibrous connective tissue (*1*), forming a capsule and growing into the implant structures (*2*); (a)–(c) hematoxylin and eosin staining; (d) special Van Gieson staining; ×100

On day 30 of the experiment, there was partial biodegradation of the implanted HPO samples in the damaged area with simultaneous active formation of connective tissue. In some areas of the examined preparations, there was mild proliferation of mesenchymal tissue around the implant accompanied by proliferation of osteoblasts and osteoid formation, which was more pronounced in the experimental group than in the control group. However, the particles of tested HPO material were not included in the composition of the osteogenic tissue and were located separately from it in clusters (Figure 4).

**Figure 5:**
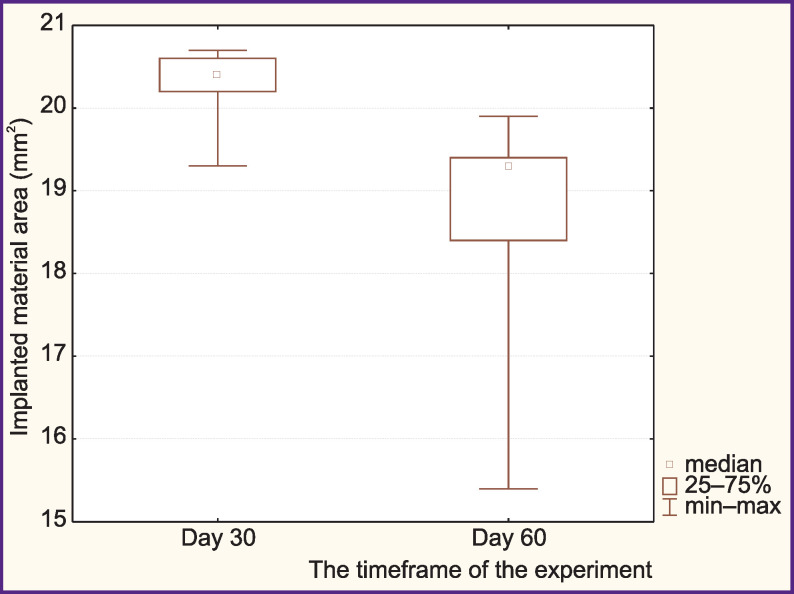
Indicator of biodegradation of HPO implant in the experiment: decreases by 22.4% by day 60, p=0.031

On day 60 after implantation of HPO samples into the bone defect, immature bone tissue was formed in the defect area. It should be noted that the number of osteogenic cells increased significantly as compared to the control group, while the bone tissue had a more organized structure. The mass of osteogenic tissue increased considerably, but the structure of bone trabeculae remained chaotic. The amount of polymer per bone tissue unit decreased by 22.4% on day 60 of the experiment as compared to day 30 (Figure 5), which may speak of material biodegradation.

**Figure 6:**
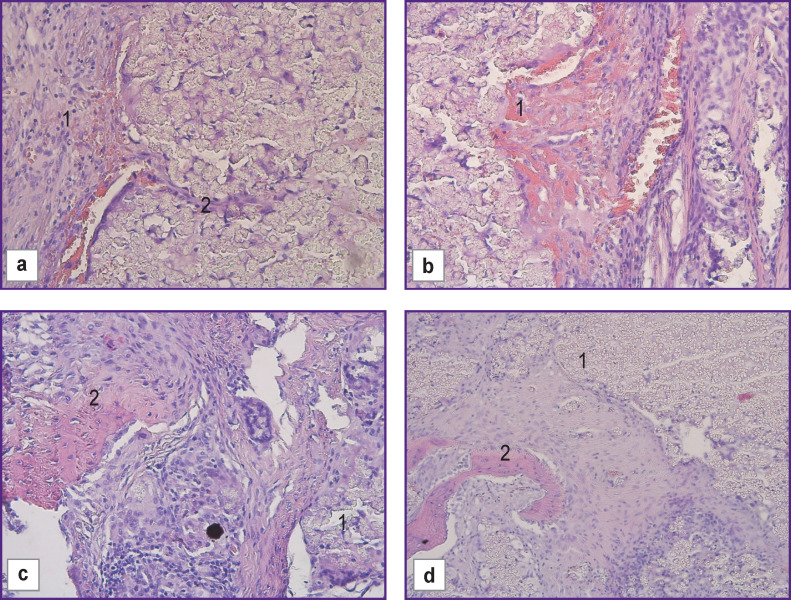
Morphological changes in the group with HPO, day 60 of the experiment: (a) granulation tissue and newly formed vessels (*1*), connective tissue growing between the implant fragments (*2*); (b) gradual growth of delicate fibrous connective tissue and newly formed vessels over the implant structures (*1*); (c) there is proliferation of osteogenic cells around the fine-granular structures of the material (*1*), their transformation into osteoblasts and formation of new bone tissue consisting of bone trabeculae with capillary vessels forming in the intertrabecular spaces (*2*); (d) new bone tissue consisting of bone trabeculae is beginning to form (*2*) in the implant area (*1*); hematoxylin and eosin staining; ×100

The newly formed bone tissue had a loose structure and multidirectional plates. The polymer particles formed aggregates consisting of a large number of small particles. Mesenchymal cells and capillary-type blood vessels surrounded some of the aggregates. The HPO did not interact with the surrounding cells and did not induce an inflammatory response (Figure 6).

Formation of connective tissue in the implant area had certain differences in animals of the control and experimental groups. In the control group, a connective tissue capsule was formed around the implant area, while in the experimental group, there was also observed growth of connective tissue fibers through the implant with active formation of granulation tissue.

**Figure 7:**
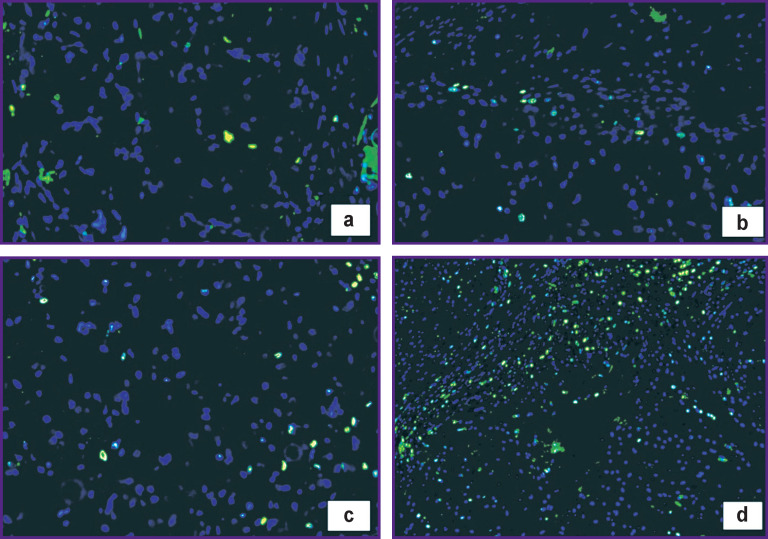
Cell nuclei in the area of allograft implantation (a), (c) and HPO implantation (b), (d): (a), (b) 30 days; ×200; (c), (d) 60 days; ×100. Fluorescence microscopy: Ki-67-positive cell nuclei are stained green, Ki-67-negative cell nuclei are blue. Green staining — antibodies to Ki-67; Goat Anti-Mouse IgG H+L, Alexa Fluor 488; blue staining — Hoechst 3334 fluorochrome (excitation wavelength 377 nm, emission 447 nm)

**Figure 8:**
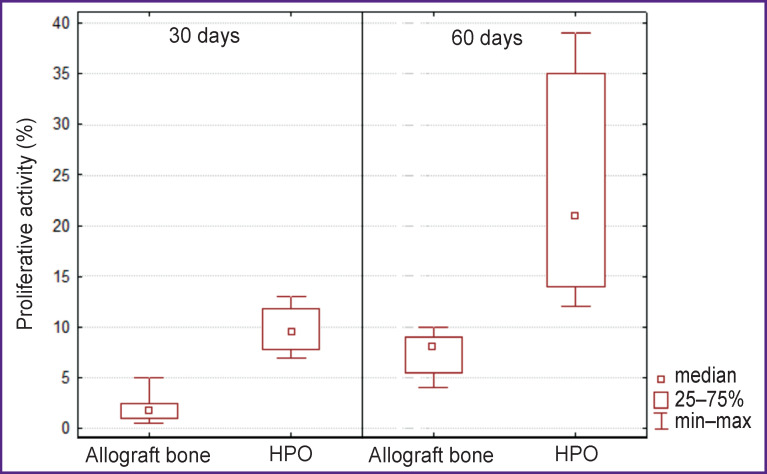
Cell proliferative activity in the studied tissues

It is interesting to compare proliferative activity indices Ki-67 indicating regeneration process activity in the control and experimental groups (Figures 7, 8).

In the control group, the proportion of Ki-67-positive cells increased during the experiment from 1.75% (4 weeks) to 8% (8 weeks). In the experimental group, the proportion of Ki-67-positive cells was initially at a higher level (9.5%) and increased to 21% by day 60, which suggested the ability of the HPO to activate cell proliferation processes.

Analysis of the data obtained showed that proliferative activity of cells that migrated and functioned in the area of HPO implantation on week 4 of the experiment was more than 5 times higher than in allograft implantation area (p=0.0002), while on week 8 it was 2.6 times higher (p=0.0058).

Thus, the results obtained demonstrated certain features of bone tissue regeneration in the experimental group animals as compared with the control. Regeneration processes in the experimental group were distinguished by active formation of delicate fibrous connective tissue with capillary vessels growing actively into it, formation of granulation tissue, and the onset of direct osteogenesis even at the early stages of the experiment (30 days). In the control group, there was observed formation of connective tissue capsule and indirect osteogenesis with cartilage tissue formation, which increased the healing time. Proliferative activity in this group was five times lower than in the experimental one even at the early stages of the experiment (30 days). By the end of the experiment, there was observed a decrease in HPO volume in the implantation area as compared with the volume on week 4 of the experiment.

When using various replacement materials, the details of bone defect restoration depend both on the material itself and on the experimental model: the type of animal, the localization of the experimental bone defect, etc. [21–23]. As a rule, histological studies of interaction between materials and bone tissue are intended to assess inflammatory reactions, severity of vascularization, formation, and maturation of new bone taking into account the area around the implants [24].

In our study, no signs of pronounced inflammation were recorded in the defect area in any group, which is one of the most important indicators of suitability of materials for use in bone grafting [25]. One more fact confirming the positive effect of interaction between the materials used and the wound bed was the revealed formation of new capillary-type vessels, which is indicative of the effective angiogenic response of the native tissue to the implanted materials. Sufficient vascularization ensures adequate supply and exchange of nutrients, which is vitally important for cell migration, survival, and growth, as well as for tissue remodeling [26]. Marked migration and proliferation of mesenchymal cells and the onset of direct osteogenesis following implantation of HPO material indicated the positive effect of this material.

It was found that fragments of both bone allograft and HPO remained in the implantation area until the end of the experiment (60 days). At the same time, the polymer content in the defect area decreased statistically significantly (by 22.4%). Retention of implant fragments or graft fragments in the defect area depends on the composition and structure of the material and may have no interference in the process of bone remodeling as biocompatibility of the material enables new bone elements to form around its fragments [27].

Since the developed material consisted of two polymers, one of which was biodegradable and the other was more stable, we can assume that the recorded decrease in the volume of the implant material in the experimental defect was associated both with polylactide biodegradation and with destruction or washing a part of polyoligomer out by tissue fluid. At the same time, the possibility of changing the ratio of HPO components can further allow changing its properties and adapting them to meet the specific requirements for restoration of bone defects of different localization.

## Conclusion

Hybrid polyoligomer material under investigation has demonstrated biocompatibility when interacting with human cells in an *in vitro* model: when cultured on its surface, the cells spread well, retained their typical morphology and viability, it was non-toxic. The absence of inflammation signs by week 8 of the *in vivo* experiment, marked neoangiogenesis, active proliferation of mesenchymal cells in the experimental defect area of animals, and the onset of new bone formation also confirm biocompatibility of the hybrid material.

The data obtained make it possible to consider the developed hybrid polyoligomer material advantageous for further research as a bone tissue substitute.
